# Control of Neural Stem Cell Survival by Electroactive Polymer Substrates

**DOI:** 10.1371/journal.pone.0018624

**Published:** 2011-04-11

**Authors:** Vanessa Lundin, Anna Herland, Magnus Berggren, Edwin W. H. Jager, Ana I. Teixeira

**Affiliations:** 1 Department of Cell and Molecular Biology, Karolinska Institute, Stockholm, Sweden; 2 Organic Electronics, Department of Science and Technology, Linköping University, Norrköping, Sweden; University of Pennsylvania, United States of America

## Abstract

Stem cell function is regulated by intrinsic as well as microenvironmental factors, including chemical and mechanical signals. Conducting polymer-based cell culture substrates provide a powerful tool to control both chemical and physical stimuli sensed by stem cells. Here we show that polypyrrole (PPy), a commonly used conducting polymer, can be tailored to modulate survival and maintenance of rat fetal neural stem cells (NSCs). NSCs cultured on PPy substrates containing different counter ions, dodecylbenzenesulfonate (DBS), tosylate (TsO), perchlorate (ClO_4_) and chloride (Cl), showed a distinct correlation between PPy counter ion and cell viability. Specifically, NSC viability was high on PPy(DBS) but low on PPy containing TsO, ClO_4_ and Cl. On PPy(DBS), NSC proliferation and differentiation was comparable to standard NSC culture on tissue culture polystyrene. Electrical reduction of PPy(DBS) created a switch for neural stem cell viability, with widespread cell death upon polymer reduction. Coating the PPy(DBS) films with a gel layer composed of a basement membrane matrix efficiently prevented loss of cell viability upon polymer reduction. Here we have defined conditions for the biocompatibility of PPy substrates with NSC culture, critical for the development of devices based on conducting polymers interfacing with NSCs.

## Introduction

The stem cell microenvironment holds the balance between stem cell proliferation and differentiation, critical during embryonic development and tissue regeneration. Microenvironmental cues include chemical signals originating from the extracellular matrix, soluble growth factors and cell-cell interactions. In addition, we and others have previously shown that the physical nature of the microenvironment provides mechanical [1], [2] and topographical [3], [4] cues to cells. Conducting polymer-based devices have the potential to afford a high degree of control over the physical and chemical stimuli sensed by stem cells and thereby provide new insights into the mechanisms that regulate stem cell state and fate [5], [6]. Additionally, manipulating the stem cell microenvironment *in vivo* through conducting polymer scaffolds holds promise to improve the outcomes of stem cell therapy.

Neural stem cells are self-renewing, multipotent cells present in the developing and adult central nervous system (CNS) that have the ability to differentiate into all neural lineages. Neural stem cells isolated from developing embryos at embryonic day 15.5 (fetal NSCs) have proven valuable for understanding CNS development and neurodevelopmental disorders [7]. Further, neural stem cells derived from pluripotent embryonic stem cells (ESC-NSCs) constitute a readily available progenitor population that has the potential to be instrumental in cell therapy in the CNS [8]. However, the development of methods to control the NSC microenvironment *in vitro* and *in vivo* is required to realize the promise of NSCs as a tool for understanding embryonic development and for cell therapy.

Reversible switching between redox states of conducting polymers can dynamically control bulk properties such as volume, conductivity and mechanical properties [9]. Additionally, the surface tension and surface chemistry of conducting polymers can be tailored by the synthesis method and the redox state of the polymers [10], [11]. The counter ion incorporated upon synthesis of conducting polymers has critical significance for their physical and chemical properties and their biocompatibility [12].

We investigated the biocompatibility of neural stem cells of fetal and embryonic origin with polypyrrole (PPy) substrates containing four anionic dopants of varying molecular weight and chemical character: dodecylbenzenesulfonate (DBS), tosylate (TsO), perchlorate (ClO_4_) and chloride (Cl). These dopants were selected due to previous extensive characterization and well-known suitability to form PPy films in devices [12]–[19]. However, little is known about the stem cell biocompatibility of these materials in electroactive applications. PPy(DBS) supported neural stem cell maintenance and differentiation along all neural lineages. Electrical reduction of the polymer critically decreased cell viability, essentially constituting an electronic switch for neural stem cell survival. Viability of NSCs upon reduction of the polymers was effectively rescued by coating the polymers with a basement membrane matrix. In this study, we have defined the working parameters for the development of devices based on conducting polymers interfacing with neural stem cells.

## Materials and Methods

### Ethics Statement

Animals were treated in accordance with institutional and national guidelines and ethical permission for this work was granted by Northern Stockholm's animal research ethics committee (Norra Djurförsökningsnämnd, permit number N79/08).

### Materials

Pyrrole monomer solution was acquired from Sigma-Aldrich, distilled upon arrival, and stored at −20°C until use. Sodium dodecylbenzenesulfonate (NaDBS) was obtained from TCI Europe, sodium p-toluenesulfonate (NaTsO) and sodium perchloride (NaClO_4_) both from Sigma-Aldrich, and NaCl from Merck. Commercial premixed electroplating solutions Neutronex 309A and 309B were acquired from Enthone. Poly-L-lysine, fibronectin, PBS, gelatin, Triton X-100, bovine serum albumin (BSA), HEPES and NaHCO_3_ were obtained from Sigma-Aldrich. DAPI, HBSS, DMEM:F12, B27 and Geltrex^TM^ Reduced Growth Factor Basement Membrane Matrix were bought from Invitrogen. Fibroblast growth factor-2 (FGF2) and epidermal growth factor (EGF) were purchased from R&D systems. EuroMed-N was from EuroClone. The following primary antibodies and dilutions were used: mouse anti-Nestin from BD Biosciences Pharmingen (1∶500), rabbit anti-glial fibrillary acidic protein (GFAP) from Dako (1∶500), mouse anti-neuronal class III ß-tubulin (Tuj1) from Nordic Biosite (1∶500), rat anti-myelin basic protein (MBP) from Chemicon (1∶250), rabbit anti-fibronectin from Sigma-Aldrich (1∶500) and rabbit anti-brain lipid-binding protein (BLBP) from Millipore (1∶100). The secondary antibodies used were species-specific conjugated Alexa-488 and Alexa-594 (1∶500) and were obtained from Molecular Probes.

### Preparation and characterization of PPy samples

**Figure 1:**
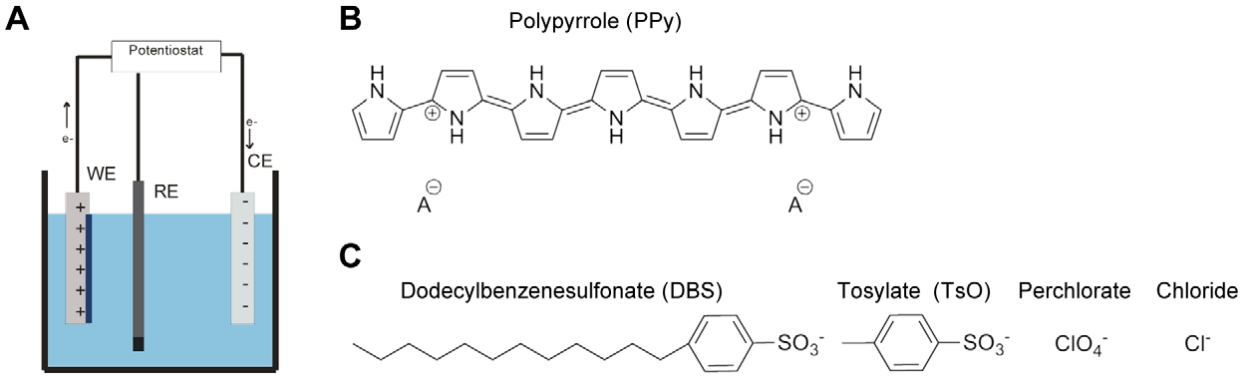
Fabrication of PPy films. (A) Schematic illustration of electrosynthesis setup. PPy was deposited onto gold coated silicon wafers using a constant potential, for each of the four counter ions. (B) Chemical structure of PPy. A^−^ represents the counter ion incorporated in the polymer to maintain charge neutrality. (C) Chemical structures showing the four different counter ions incorporated in the polymerization of PPy: DBS, TsO, ClO_4_ and Cl.

Standard 100 mm silicon wafers were coated with a thermally evaporated 5 nm chromium adhesion layer and a 100 nm gold layer. Using standard photolithography, a photoresist pattern was transferred onto the wafer comprising rectangular openings of 8×10 mm^2^ in the photoresist for PPy deposition. Hereafter, PPy was electrosynthesized on the gold exposed in the photoresist using an Ecochemie microAutolabIII (Metrohm Autolab) potentiostat (Figure 1A). The electrolyte was an aqueous solution of 0.1 M pyrrole monomers and 0.1 M NaDBS, NaTsO, NaClO_4_, or NaCl. A constant potential of 0.57 V, 0.65 V, 0.60 V or 0.70 V vs. Ag/AgCl was applied for NaDBS, NaTsO, NaClO_4_ or NaCl, respectively. (In the case of NaCl, the potential was set at 0.75 V for the first 120 sec and thereafter reduced to 0.70 V for the remainder of the polymerization time). Polymerization was stopped when a charge of 6 C ( = 0.27 C/cm^2^) was reached. For the samples used in the electrical activation experiments, an adhesion layer of rough, electroplated gold was electrochemically deposited on the gold substrate before electrosynthesizing PPy [20]. A diluted commercial gold electroplating solution of 10 ml Neutronex 309 part A, 300 ml Neutronex 309 part B and 190 ml H_2_O was used. A potential of V  =  −0.7 V vs. Ag/AgCl was applied for 120 sec, followed by V  =  −0.9 V vs. Ag/AgCl for 240 sec. After the polymerization, the wafers were rinsed with DI water and the photoresist was stripped using acetone, followed by a rinse with DI water. Next, the wafers were diced into the final sample size of 10×20 mm^2^ using a scriber. Thickness and surface roughness measurements were performed with a Dektak 3ST surface profiler (Veeco Instruments). The PPy substrates were sterilized under UV for 10 min and then soaked overnight, first in cell culture grade water, then in PBS at 37°C.

### Electrical reduction of substrates

The PPy films were stimulated by a linear ramping of 0.02 V/sec, followed by a constant potential of V  =  −0.9 V vs. Ag/AgCl for 120 sec. PPy was reduced in pre-warmed N2 media and the cells were allowed to recover in the incubator for 5 hrs after the electrical stimulation, before they were fixed and stained. For PPy substrates reduced prior to cell seeding, the NSCs were seeded onto the polymer films immediately after reduction. The reduction of PPy(DBS) occurs according to the reaction [16]:

PPy^+^(DBS^−^) + M^+^(aq) + e^−^ ⇔ PPy^0^(DBS^−^M^+^), where M^+^ is a small cation present in the cell culture medium. The smaller counter ions are mobile and reduction of the polymer follows the reaction:

PPy^+^(A^−^) + e^−^ ⇔ PPy^0^ + A^−^(aq), where A^−^ represents the cations TsO^−^, ClO_4_^−^ and Cl^−^.

### Cell culture

#### Fetal NSC Culture

Primary neural stem cells were obtained by dissecting and dissociating cerebral cortices from Sprague-Dawley rat embryos at embryonic day 15.5. The fetal cells were plated on 100 mm dishes coated with 15 µg/ml poly-L-ornithine and 1 µg/ml fibronectin. Cells were expanded in serum-free DMEM:F12 medium with N2 supplement and 10 ng/ml FGF2 to maintain the proliferative state [21]. The NSCs were used at passage two in all experiments and cells were passaged by dissociation in HBSS, HEPES and NaHCO_3_. The cells were seeded on PPy substrates at 16,000 cells/cm^2^ and cultured for two days. For gelatin treatments, substrates were incubated with 0.1% gelatin for 1 hour at 37°C, prior to coating with poly-L-ornithine and fibronectin. For the BMM coating of PPy, Geltrex^TM^ Reduced Growth Factor Basement Membrane Matrix was thawed overnight on ice at 4°C. PPy substrates were coated with 100 µl of Geltrex solution at approximately 9 mg/ml and incubated at 37°C for 60 min. For thin BMM coating, PPy substrates were incubated with 50 µl of Geltrex solution at approximately 4.5 mg/ml. For *in vitro* differentiation, cells were grown for seven days, in the presence or absence of FGF2.

#### ES Cell-Derived NSC (ESC-NSC) Culture

A neural stem cell line was obtained by differentiation of mouse ES cells (CSL510 clone 9) as previously described by Pollard *et al*. [22]. Briefly, neural progenitor differentiation was induced by culturing ES cells in serum-free N2B27 medium for 8 days in 100 mm dishes coated with 0.1% gelatin. Thereafter, cells were replated onto low attachment culture plates in fresh media supplemented with 10 ng/ml EGF and FGF2. After 4 days, the newly formed cell aggregates were plated in gelatinized dishes in NS basal medium with N2 supplement and EGF and FGF2. After cell attachment, cells were passaged using trypsin 3–5 times to obtain a stable neural stem cell line. The cells were seeded on PPy uncoated or gelatinized, at 16,000 cells/cm^2^ and cultured for two days at passage 10–15.

### Immunocytochemistry and nucleofection

For immunocytochemistry, cells were rinsed with PBS and fixed in 10% formalin for 15 min at room temperature (RT). The samples were washed in PBS/0.1% Triton X-100 three times for five min before incubation overnight at 4°C with the primary antibody in PBS/0.1% Triton X-100/1% BSA. The cells were washed six times for five min in PBS/0.1% Triton X-100, and then incubated with secondary antibody (1:500) in PBS/0.1% Triton X-100/1% BSA 1 hour at RT in the dark. Finally, the samples were rinsed three times with PBS and counterstained with 4′,6-diamidino-2-phenylindole (DAPI). Images were acquired using a fluorescent Zeiss Axioskop2 microscope and a Zeiss AxioCam MRm camera with Axiovision Rel 4.6 software.

For live detection of cells on BMM gels, NSCs were transfected with 1 µg pmaxGFP using a Nucleofector device and Rat NSC Nucleofector Kit according to the vendor's protocol (Amaxa, Lonza), then seeded onto the BMM coated PPy(DBS).

### Real-time quantitative PCR

Real-time quantitative PCR was performed on the fetal NSCs after 7 days in culture. RNA was extracted using RNeasy and contaminating DNA was removed using RNase free DNase kit (Qiagen). cDNA was synthesized from 200 ng of RNA using High Capacity cDNA Reverse Transcription kit (Applied Biosystems). Real-time PCR was performed using Platinum SYBR Green qPCR SuperMix UDG (Invitrogen) and the 7300 Real Time PCR System with the following thermo cycling program: 50°C for 2 min, 95°C for 2 min and 40 cycles of 95°C for 15 sec and 60°C for 30 sec, and finally 95°C for 15 sec, 60°C for 1 min and 95°C for 15 sec. Each sample was run in triplicates and samples without reverse transcriptase were used as controls. The standard curve method was used to evaluate mRNA levels relative to TATA binding protein (TBP), which was the endogenous control. Primers are available upon request.

### Statistical analysis

All statistical analysis was done in GraphPad Prism 5, using significance levels at *P < 0.05, **P < 0.01 and ***P < 0.001. Details of statistical analysis for each experiment were included in the figure legends.

## Results

### Substrate fabrication and characterization

Photolithographically patterned PPy films (Figure 1B) were potentiostatically synthesized on gold substrates with four common dopants: dodecylbenzenesulfonate (DBS), tosylate (TsO), perchlorate (ClO_4_) and chloride (Cl) (Figure 1C). The resulting films of PPy(DBS), PPy(TsO) and PPy(Cl) were of similar thickness. PPy(DBS) and PPy(TsO) films showed low roughness, whereas PPy(Cl) was mostly smooth, but did show spikes or particles on/in the film. However, PPy(ClO4) films were thicker and rougher than all other films, showing larger peaks of high amplitude on the surface (Table 1). Similar correlation between film thickness and surface roughness was previously reported for PPy films [12], [23], [24].

**Table 1:** Characterization of PPy films.

	d (µm)	R_a_ (nm)
PPy(DBS)	1.87±0.29	10.0±0.55
PPy(TsO)	0.98±0.16	16.9±6.5
PPy(ClO_4_)	7.16±0.91	247±66.9
PPy(Cl)	0.75±0.15	106±30.0

Thickness (d) and surface roughness (R_a_) was measured with a surface profiler.

### Effect of doping ion on the survival of NSCs grown on polypyrrole

**Figure 2:**
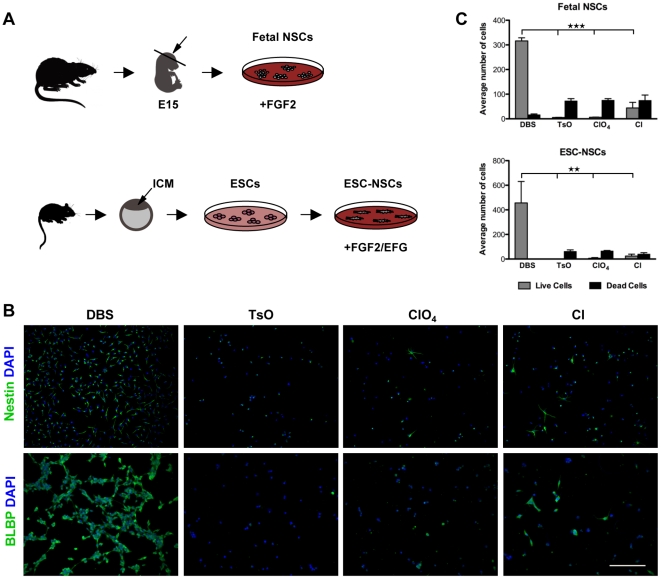
NSC viability on PPy depended on the counter ion incorporated in the conducting polymer. (A) Alternative methods used to obtain NSC cultures: fetal NSCs were derived from developing cerebral cortices from rats and kept in a proliferative state by the addition of FGF2, whereas ESC-NSC were derived from previously isolated ESCs from mice and kept multipotent in the presence of FGF2/EGF. ICM  =  inner cell mass. (B) Fetal NSCs and ESC-NSCs grown on PPy(DBS) for 2 days in N2 media supplemented with FGF2 or FGF2/EGF showed immunoreactivity for the NSC markers Nestin and BLBP, respectively. TsO, ClO_4_ and Cl as counter ions in PPy compromised cell viability. (C) Quantification of the average number of live and dead cells in (B) as calculated from five random 10x images from three separate experiments. PPy(DBS) surfaces showed high NSC viability, whereas surfaces with the three smaller ions, TsO, ClO_4_ and Cl, had none or few live cells. Fetal NSCs and ESC-NSCs were very similar in their response to the PPy doping ion. 1-Way ANOVA analysis of variance was used followed by Tukey's multiple comparison test. The scale bar represents 200 µm.

Fetal neural stem cells (fetal NSCs) harvested from the developing rat cerebral cortex at embryonic day 15.5 and cultured in N2 medium supplemented with fibroblast growth factor-2 (FGF2) remain in a proliferative and multipotent state *in vitro* [21] (Figure 2A). To investigate the effect of doping ion on the biocompatibility of PPy films with neural stem cell culture, fetal NSCs were grown on PPy(DBS), PPy(TsO), PPy(ClO_4_) and PPy(Cl). The films were used in the pristine state, *i.e*. no potential was applied. All PPy substrates were pre-coated with poly-L-ornithine and fibronectin, a standard procedure in culturing fetal NSCs as adherent monolayers. Fetal NSCs cultured on PPy(DBS) films for 2 days in the presence of FGF2 maintained the neural stem cell state, indicated by immunoreactivity for the intermediate filament protein Nestin (Figure 2B). In addition, cell viability was high on PPy(DBS) substrates with few cells showing pyknotic nuclei, a hallmark of cell death (Figure 2C). In contrast, NSC viability was severely compromised on the PPy surfaces doped with the three smaller ions, TsO, ClO_4_ and Cl. Few Nestin positive cells were present on the PPy(TsO) and PPy(ClO_4_) films (Figure 2B) and indeed most cells showed pyknotic nuclei on these substrates (Figure 2C). On the PPy(Cl) films, the level of dead cells was similar to PPy(TsO) and PPy(ClO_4_) but live cells could also be observed, albeit at a significantly lower level than on the PPy(DBS) surfaces (Figure 2C).

Cells with neural stem cell properties can be derived from embryonic stem cells *in vitro* [22], and are an alternative source of NSCs that circumvent the requirement of primary isolation present in fetal NSCs. These cells (ESC-NSCs) can be maintained indefinitely in a neural stem cell state when cultured in medium containing FGF2 and epidermal growth factor (EGF), and show immunoreactivity for brain lipid-binding protein (BLBP), a protein expressed endogenously by NSCs with radial glial characteristics (Figure 2A). ESC-NSC showed a remarkably similar correlation between PPy doping ion and cell viability compared to fetal NSCs. ESC-NSCs also showed high cell viability on PPy(DBS) whereas ESC-NSCs grown on PPy containing the three smaller dopants, TsO, ClO_4_ and Cl, showed little or no viability (Figure 2C). Significantly, Retinal Pigmented Epithelial (RPE) cell adhesion and proliferation was similar on PPy(Cl) and PPy(ClO_4_) substrates compared to PPy(DBS) (Figure S1). This result is consistent with previous reports showing the biocompatibility of PPy surfaces with a range of cell types [23]-[25], highlighting the unique sensitivity of NSCs to the counter ion incorporated in conducting polymer-based cell culture substrates.

Differences in conformation of adsorbed fibronectin between oxidized and reduced substrates, composed of the conjugated polymer poly(3,4-ethylenedioxythiophene) (PEDOT) doped with tosylate, were previously shown to direct epithelial cell attachment and viability [26]. To determine whether the conformation of the adsorbed fibronectin layer was responsible for the observed dependence of NSC viability on PPy doping ion, we immunolabeled the fibronectin adsorbed onto the PPy substrates (Figure S2). No significant differences were observed in the average fluorescence intensity of the immunolabeled fibronectin adsorbed onto the substrates doped with DBS, TsO, ClO_4_ and Cl. Additionally, fetal NSCs and ESC-NSCs showed the same correlation between cell viability and PPy doping ion when the substrates were pre-coated with gelatin (Figure S2). Together, these results suggest that the adsorbed protein layer was not a critical mediator of the poor biocompatibility of PPy doped with TsO, ClO_4_ or Cl with NSC culture.

### NSC maintenance and differentiation on PPy(DBS)

**Figure 3:**
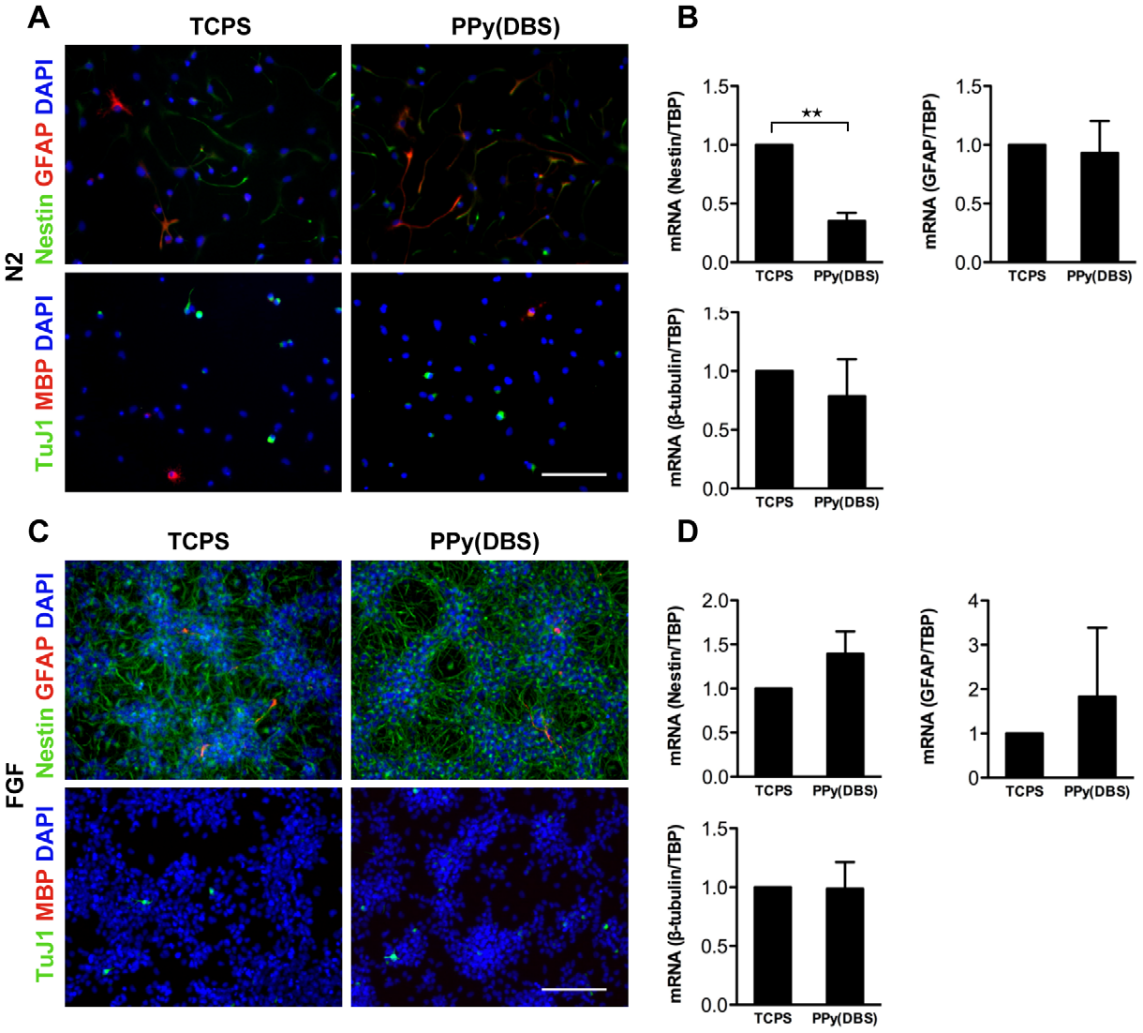
PPy(DBS) supported long term maintenance and differentiation of fetal NSCs. (A) In the absence of FGF2, fetal NSCs differentiated into astrocytes (GFAP positive); neurons (marked with Tuj1) and oligodendrocytes (labeled for MBP). PPy(DBS) supported NSC differentiation over 7 days in N2 conditions, similar to TCPS. (B) mRNA expression levels analyzed by real-time PCR showed comparable levels of Nestin, GFAP and ß-tubulin relative to TBP for NSCs when cultured on PPy(DBS) and TCPS for 7 days in N2 media supplemented with FGF2, validated by their immunoreactivity for Nestin. (D) Real-time quantitative PCR results showed similar mRNA levels for Nestin, GFAP and ß-tubulin relative to TBP for fetal NSCs grown on TCPS and PPy(DBS). Student's two-tailed t-test was used. The scale bar represents 100 µm.

Since the PPy(DBS) surfaces best supported the survival of NSCs, PPy(DBS) was selected for all subsequent experiments. Withdrawal of the mitogen FGF2 from the cell culture medium causes fetal NSCs to exit the cell cycle and spontaneously differentiate along all neural lineages, giving rise to astrocytes, neurons and oligodendrocytes [1]. To investigate the ability of PPy(DBS) to support NSC differentiation, fetal NSCs were cultured on the polymer for 7 days in the absence of FGF2. The NSCs on PPy(DBS) differentiated into astrocytes, confirmed by glial fibrillary acidic protein (GFAP) immunoreactivity; neurons, labeled with an antibody against neuron-specific class III ß-tubulin (TuJ1); and oligodendrocytes identified by myelin basic protein (MBP) labeling (Figure 3A). Real-time quantitative PCR (qPCR) showed that the pattern of NSC differentiation on PPy(DBS) was comparable to differentiation on standard tissue culture polystyrene (TCPS) substrates (Figure 3B). Specifically, the levels of mRNA expression of GFAP and class III ß-tubulin were similar for cells differentiated on PPy(DBS) and TCPS (Figure 3B). MBP mRNA was not detected in this assay, likely due to the expected low levels of oligodendrocytic differentiation. To investigate the biocompatibility of PPy(DBS) upon extended culture, fetal NSCs were cultured for 7 days in N2 medium containing FGF2. NSCs proliferated and maintained the stem cell state, validated by Nestin staining (Figure 3C). A small number of the cells expressed the astrocytic marker GFAP or the neuronal marker TuJ1, at levels comparable to fetal NSCs grown on TCPS (Figure 3D). These data validate PPy(DBS) as an appropriate substrate for fetal NSC maintenance and differentiation.

### NSC viability upon reduction of PPy(DBS)

**Figure 4:**
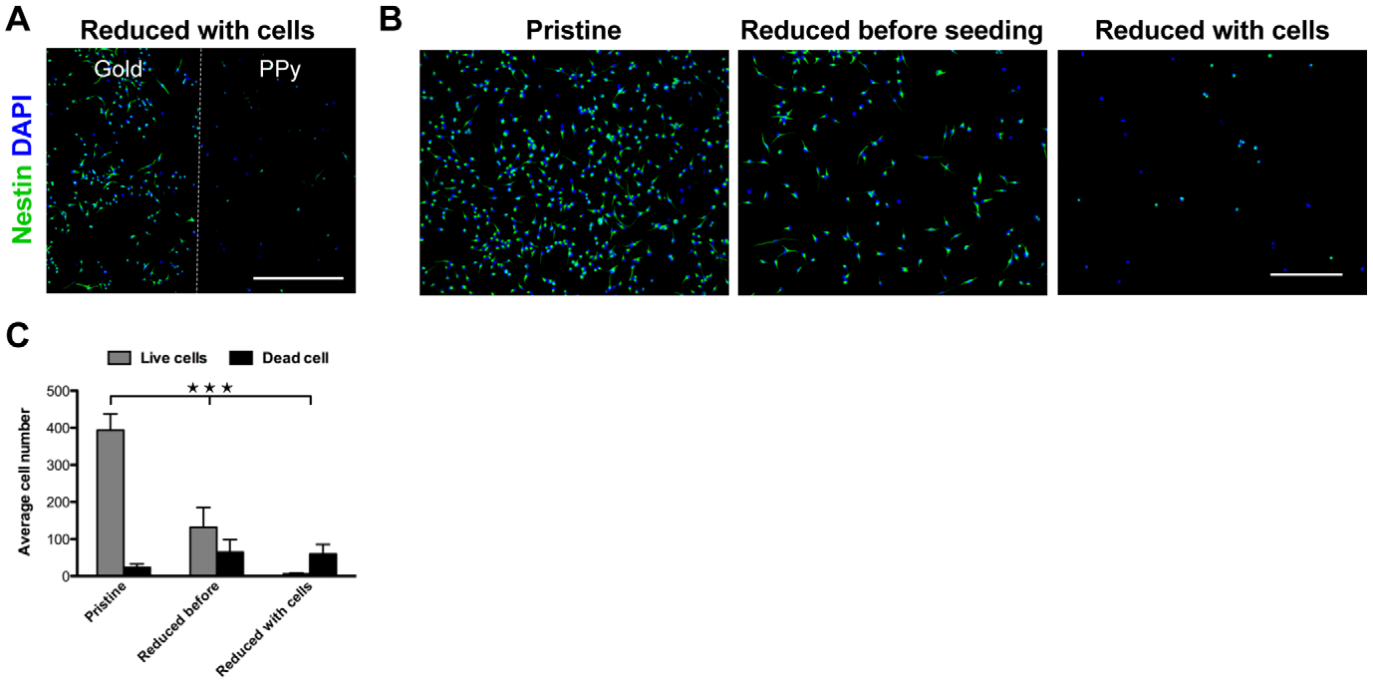
Electrical reduction of PPy(DBS) caused alterations in polymer surface properties, leading to impaired cell viability. (A) Fetal NSCs were cultured on the PPy(DBS) films for 2 days before electrical reduction of the substrates at -0.9 V vs. Ag/AgCl. Reduction of PPy(DBS) caused cell death and detachment from the substrate. However, NSC viability on the gold coated areas of the substrates was not impaired. The dashed line indicates the gold/PPy(DBS) border. The scale bar represents 500 µm. (B) NSC viability on substrates reduced prior to cell seeding was lower than on substrates in the pristine state. Reduction of the polymer with live cells led to an even larger decrease in cell viability. The scale bar represents 200 µm. (C) Quantification of the average number of live and dead cells in (B) as calculated from five random 10x images from three separate experiments. 1-Way ANOVA analysis of variance followed by Tukey's multiple comparison test was used.

Electrical reduction of PPy(DBS) causes cations to enter the polymer in order to maintain charge neutrality. The influx of ions leads to an expansion of the polymer, a process which has been extensively exploited in the fabrication of microactuators [27]. To investigate the effect of electrical reduction on the viability of NSCs cultured on PPy substrates, PPy(DBS) films seeded with fetal NSCs were reduced by applying a voltage of -0.9 V for 2 minutes. These conditions have previously been shown to be sufficient to attain full reduction of the polymer [19]. NSC viability following PPy(DBS) reduction was poor and most cells detached from the polymer. Importantly, NSCs grown on the gold coated areas of the same substrates showed no significant loss of viability upon application of the reducing potential to the gold electrodes (Figure 4A), suggesting that the surface potential *per se* is not responsible for the observed loss of cell viability.

The reduction of PPy(DBS) has previously been shown to cause a significant decrease in the surface energy of the polymer [11]. Furthermore, reduction of another conducting polymer, PEDOT(TsO), has previously been shown to inhibit the attachment of a neural stem cell line (c17.2) [5], indicating that the redox state of the polymer, and thus the surface energy, can affect cell adhesion and viability. To investigate the role of surface properties on the loss of viability of NSCs upon PPy(DBS) reduction, NSCs were cultured on PPy(DBS) substrates that had been reduced prior to cell seeding (Figure 4B). On these surfaces, the number of viable cells dropped to approximately one third compared to pristine surfaces, suggesting that modifications in surface properties caused by the reduction of the polymer had a negative impact on cell viability. However, the changes in surface properties due to reduction of the polymer do not fully account for the observed loss of cell viability when PPy(DBS) is reduced with live cells. Indeed, the number of live cells was lower when the polymers were reduced with live cells compared to polymer reduction prior to cell seeding (Figure 4B,C).

**Figure 5:**
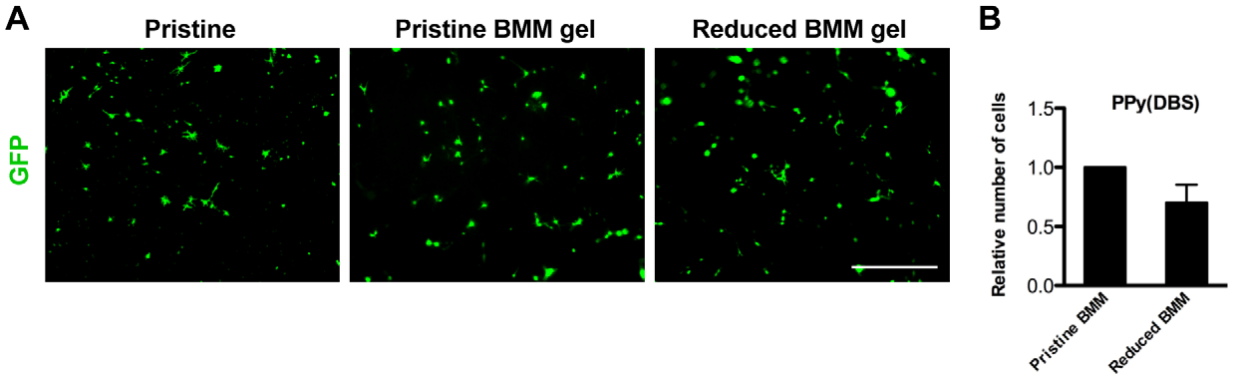
Treating PPy with a gel layer of BMM efficiently rescued cell viability upon electrical reduction. (A) NSC viability was high for cells grown on pristine PPy(DBS) coated with a BMM gel layer, comparable to NSCs on pristine PPy(DBS) without BMM. NSC viability was maintained upon reduction of PPy(DBS) coated with a BMM gel. (B) Quantification of the average number of live cells in (A) as calculated by GFP expression from five random 5x images and by Nestin expression from five random 10× images from two separate experiments. 1-Way ANOVA analysis of variance was used followed by Tukey's multiple comparison test. The scale bar represents 500 µm.

In order to increase NSC survival upon PPy(DBS) reduction, PPy substrates were coated with a gel layer composed of a basement membrane matrix (BMM). BMM treatment of pristine PPy(DBS) resulted in good cell survival, comparable to pristine surfaces without BMM (Figure 5A). Importantly, cell viability was maintained after reduction of BMM treated PPy(DBS) cultured with live cells (Figure 5B). Notably, NSCs showed poor survival upon reduction of PPy(DBS) coated with a thin layer of BMM (Figure S3). Therefore, treatment of PPy(DBS) with a thick BMM gel layer effectively rescued NSC viability during the reduction process.

## Discussion

The proper integration of microenvironmental cues with cell intrinsic genetic and epigenetic programs regulates stem cell state and fate. Control of cellular inputs by PPy has received growing attention [12], [28]. Here we show that PPy can be tailored to promote cell survival and maintenance of NSCs. We have validated these results for two different NSC systems: one originating from rat embryonic cortices, *i.e*. a primary cell culture; the other derived from mouse ES cells, *i.e*. a NSC line. The two NSC systems show remarkably comparable results, confirming a consistent pattern of PPy biocompatibility with NSCs.

PPy(DBS) shows very good biocompatibility with NSCs, indistinguishable from standard tissue culture polystyrene substrates. Doping polypyrrole with any of the smaller counter ions tested, TsO, ClO_4_ and Cl, caused a dramatic decrease in stem cell viability. It has previously been shown that the chemical composition and conductivity of PPy(Cl) and PPy(TsO) films degrade over time under physiological conditions due to leaching of the dopants [23]. We suggest that this is a driving mechanism in the observed lack of biocompatibility of PPy(TsO), PPy(ClO_4_) and PPy(Cl) with NSC culture. Importantly, we showed that RPE cell culture was compatible with PPy(ClO_4_) and PPy(Cl) substrates, in accord with a host of literature showing biocompatibility of multiple cell types with PPy substrates containing different counter ions [23], [25]. This highlights the unique requirements for biocompatibility of conducting polymer substrates with NSC culture. Significantly, coating the substrates with gelatin did not alter their biocompatibility (Figure S2), suggesting that the differences in NSC viability on PPy with the various counter ions are not mediated by differences in adsorbed extracellular matrix proteins.

Reduction of PPy(DBS) substrates with cultured live cells, led to widespread cell death. To determine whether this effect was due to changes in the surface composition of the polymer upon reduction, we reduced the polymers prior to cell seeding. This caused a significant decrease in cell viability compared to pristine surfaces. However, the decrease in cell viability observed on surfaces that were reduced with cultured live cells was larger than on surfaces reduced before cell seeding. Therefore, we conclude that mechanisms originating both from changes in surface composition of the polymers or adsorbed protein layers as well as from ion fluxes generated upon polymer reduction contributed to the observed loss of viability.

Complete rescue of cell viability upon reduction of PPy(DBS) was achieved by treating the polymer with a thick gel layer of a basement membrane matrix (BMM). Significantly, coating PPy(DBS) with a thin layer of BMM led to poor NSC survival upon polymer reduction. We propose that the BMM gel layer, with a thickness of hundreds of nanometers, creates a buffer zone, which effectively protects cells from ion fluxes during the reduction process. Further, this thick gel coating rendered the cultured cells insensitive to changes in surface composition at the conducting polymer/BMM gel interface.

In conclusion, we have shown that the biocompatibility of polypyrrole (PPy) with neural stem cells (NSCs) of fetal or embryonic origin depends on the counter ion incorporated in the polymer. PPy(DBS) was best suited for NSC culture and supported long term maintenance of the stem cell state as well as differentiation along neural lineages. In contrast, TsO, ClO_4_ and Cl were not compatible PPy counter ions for NSC culture. Moreover, electrical reduction of PPy(DBS) with NSCs lead to a loss of cell viability, which was effectively prevented by coating the polymer films with a gel layer of basement membrane matrix prior to cell culture. These observations demonstrate the potential of using conducting polymer-based devices with neural stem cells for investigations on neural development and in cell therapy.

## Supporting Information

Figure S1 **RPE cell viability was detected with phalloidin and DAPI.** Adhesion and proliferation was high on PPy(DBS), PPy(Cl) and PPy(ClO_4_) substrates but low on PPy(TsO). The scale bar represents 200 µm.
(TIF)

Figure S2 **Coating PPy substrates with fibronectin or gelatin did not alter the correlation between cell viability and PPy doping ion.** (A) Immunolabeling of fibronectin on PPy surfaces showed no correlation in fibronectin adsorption between the various counter ions. No significant differences in the average fluorescence intensity of fibronectin on the various surfaces were detected as analyzed from two separate experiments. 1-Way ANOVA analysis of variance followed by Bonferroni's multiple comparison test was used. (B) NSCs cultured for 2 days on PPy pre-coated with 0.1% gelatin. PPy(DBS) supported high viability of both fetal NSCs (upper row) and ESC-NSCs (bottom row), as detected with Nestin and BLBP, respectively. PPy doped with TsO, ClO_4_ and Cl and pre-coated with gelatin showed low cell viability. (C) Quantification of the average number of live fetal NSCs and ESC-NSCs grown on PPy surfaces without or with gelatin coating. Cell numbers were obtained from five random 10x images from three independent experiments. 1-Way ANOVA analysis of variance was used followed by Tukey's multiple comparison test. The scale bars represent 200 µm.
(TIF)

Figure S3 **Thin BMM coating of PPy(DBS) did not maintain cell viability upon polymer reduction.** NSC viability was high for cells grown on pristine PPy(DBS) with a thin BMM coating, as detected by Nestin immunoreactivity. Cell viability was compromised upon reduction of PPy(DBS) with a thin BMM coating. The scale bar represents 200 µm.
(TIF)
