# Supplementary figures and images for: Control of Neural Stem Cell Survival by Electroactive Polymer Substrates
Source: PLoS One. 2011 Apr 11;6(4):e18624. doi: 10.1371/journal.pone.0018624 (PMC3073951; doi:10.1371/journal.pone.0018624)

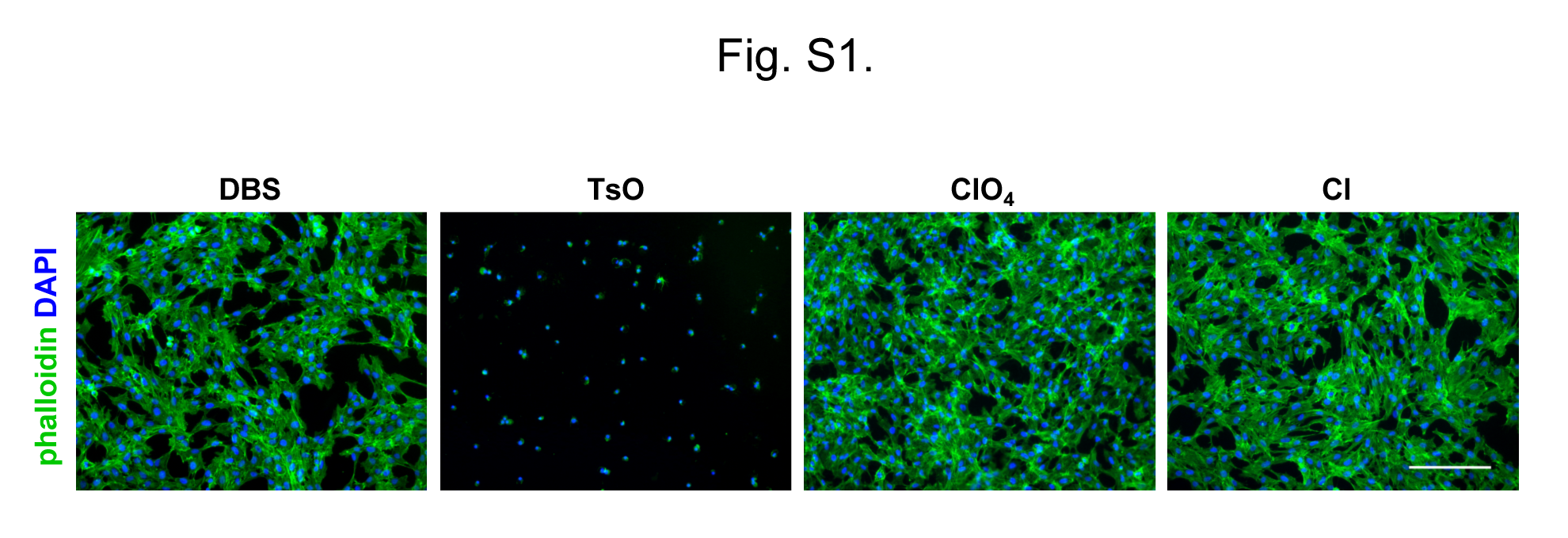

Supplement: Figure S1 — RPE cell viability was detected with phalloidin and DAPI. Adhesion and proliferation was high on PPy(DBS), PPy(Cl) and PPy(ClO4) substrates but low on PPy(TsO). The scale bar represents 200 µm. (TIF) [file pone.0018624.s001.tif]

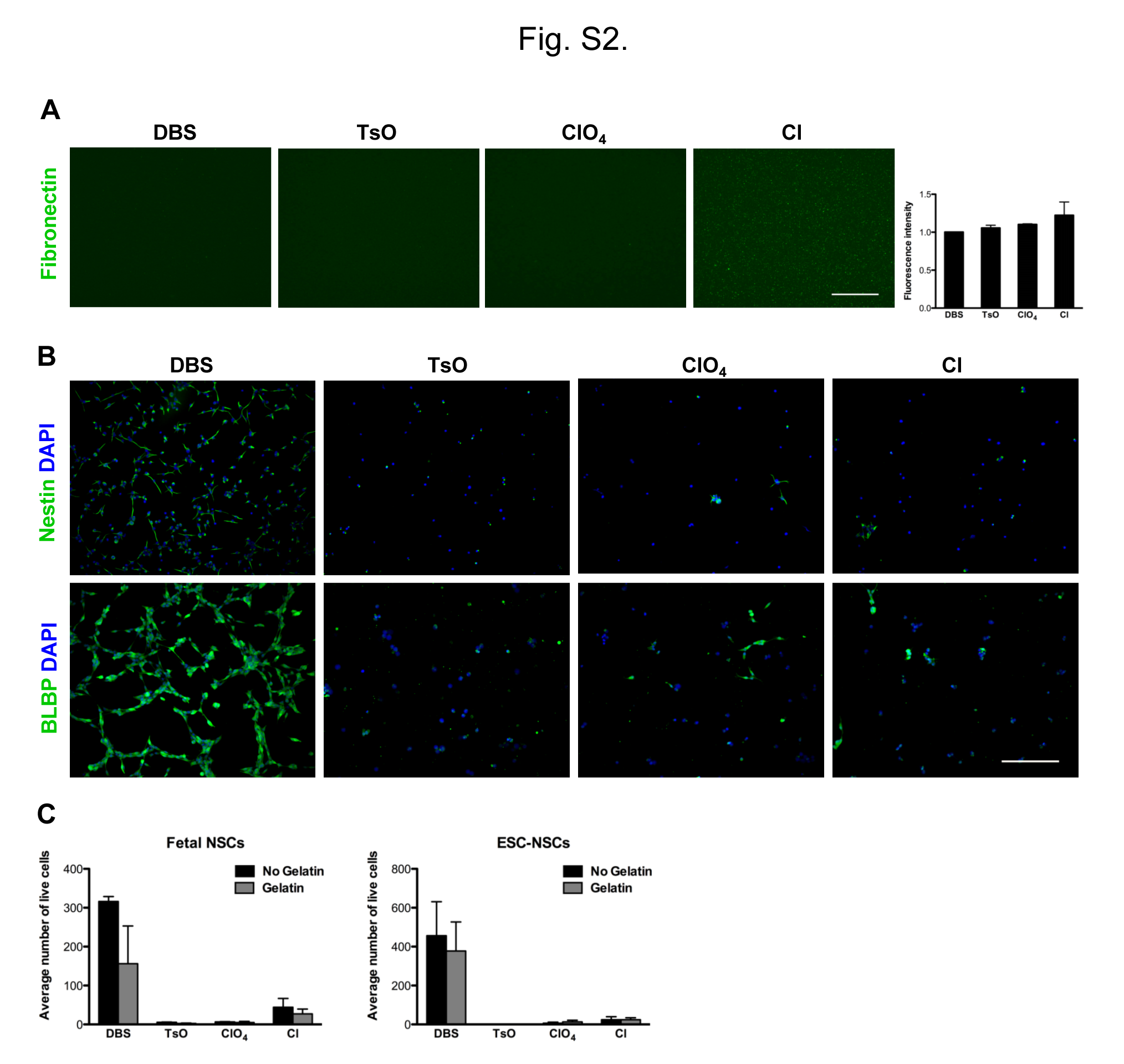

Supplement: Figure S2 — Coating PPy substrates with fibronectin or gelatin did not alter the correlation between cell viability and PPy doping ion. (A) Immunolabeling of fibronectin on PPy surfaces showed no correlation in fibronectin adsorption between the various counter ions. No significant differences in the average fluorescence intensity of fibronectin on the various surfaces were detected as analyzed from two separate experiments. 1-Way ANOVA analysis of variance followed by Bonferroni's multiple comparison test was used. (B) NSCs cultured for 2 days on PPy pre-coated with 0.1% gelatin. PPy(DBS) supported high viability of both fetal NSCs (upper row) and ESC-NSCs (bottom row), as detected with Nestin and BLBP, respectively. PPy doped with TsO, ClO4 and Cl and pre-coated with gelatin showed low cell viability. (C) Quantification of the average number of live fetal NSCs and ESC-NSCs grown on PPy surfaces without or with gelatin coating. Cell numbers were obtained from five random 10x images from three independent experiments. 1-Way ANOVA analysis of variance was used followed by Tukey's multiple comparison test. The scale bars represent 200 µm. (TIF) [file pone.0018624.s002.tif]

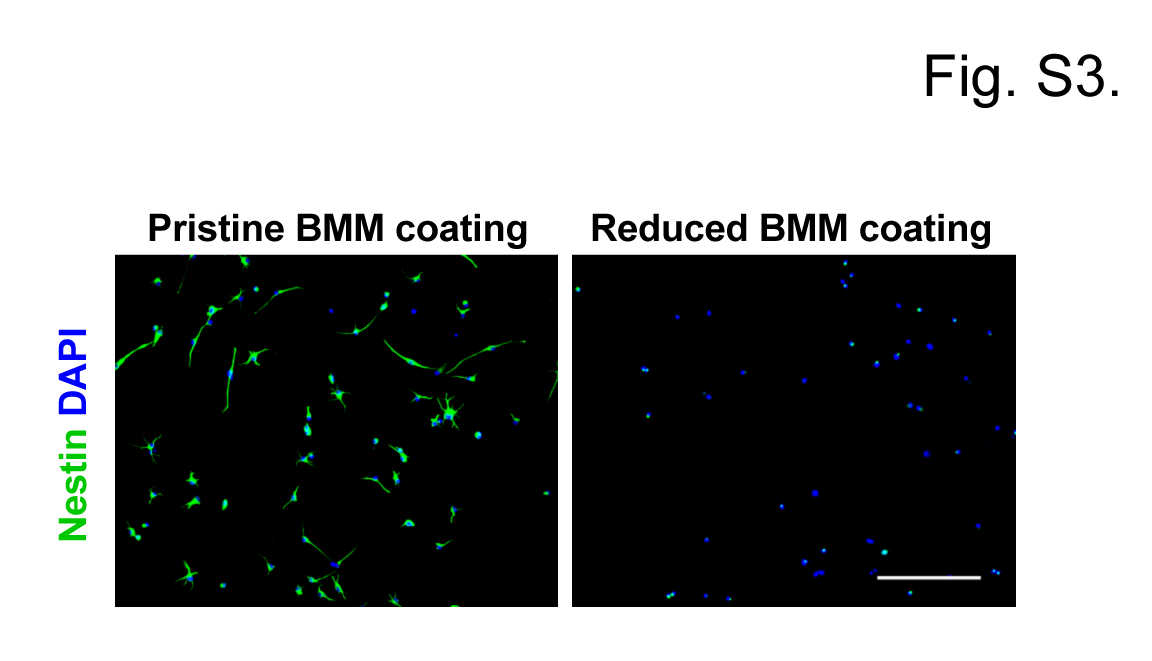

Supplement: Figure S3 — Thin BMM coating of PPy(DBS) did not maintain cell viability upon polymer reduction. NSC viability was high for cells grown on pristine PPy(DBS) with a thin BMM coating, as detected by Nestin immunoreactivity. Cell viability was compromised upon reduction of PPy(DBS) with a thin BMM coating. The scale bar represents 200 µm. (TIF) [file pone.0018624.s003.tif]
